# Awake targeted memory reactivation doesn’t work

**DOI:** 10.3758/s13421-024-01576-x

**Published:** 2024-05-14

**Authors:** Linda J. Hoffman, Julia M. Foley, Büşra Tanrıverdi, Jason Chein, Ingrid R. Olson

**Affiliations:** Department of Psychology, Temple University, 1701 N. 13th Street, Philadelphia, PA 19122 USA

**Keywords:** Replay, Reactivation, Consolidation, Vocabulary, Sleep, Second language learning, Hippocampus

## Abstract

Memories are pliable and can be biased by post-encoding information. In targeted memory reactivation (TMR) studies, participants encode information then sleep, during which time sounds or scents that were previously associated with the encoded images are re-presented in an effort to trigger reactivation of the associated memory traces. Upon subsequent testing, memory for reactivated items is often enhanced. Is sleep essential for this process? The literature on awake TMR is small and findings are mixed. Here, we asked English-speaking adults to learn Japanese vocabulary words. During a subsequent active rest phase, participants played Tetris while sound cues associated with the vocabulary words were presented. Results showed that when memories were reactivated, they were either disrupted (Experiment 1) or unaffected (Experiments 2, 3). These findings indicate that awake TMR is not beneficial, and may actually impair subsequent memory. These findings have important implications for research on memory consolidation and reactivation.

## Introduction

Consolidation is the process by which newly learned information is committed to memory for long-term storage. In a seminal study, researchers found that place cells in the CA1 region of the rodent hippocampus fired in a similar pattern during REM and slow-wave sleep as they did while the rodents were learning a spatial memory task (Skaggs & McNaughton, 1996). This marked the first evidence that a process of memory “reactivation” or “replay” during post-learning episodes may be the mechanism that governs the memory consolidation. Subsequent evidence demonstrated that the amount of replay that occurs for a given memory trace predicts later retrieval performance for that memory. That is, there is a positive relationship between the extent of the reactivation that transpires in a post-encoding period and the degree to which the replayed information is remembered (Dupret et al., 2010). Further work investigated whether the phenomenon of replay occurs only during sleep, or whether it also extends to periods of relative quiescence and rest, with several studies finding that replay also occurs spontaneously during non-sleep periods (Tambini & Davachi, 2019).

Evidence pointing to memory reactivation’s function as an agent of memory consolidation encouraged researchers to investigate whether experimental manipulations could bias reactivation such that specific memory traces were strengthened. One popular technique for doing this, targeted memory reactivation (TMR), is characterized by the sensory cueing of specific memory items, typically during post-encoding sleep in humans. Though TMR effects on subsequent memory are not always observed (Hu et al., 2020), a now large body of empirical studies have reported positive evidence that TMR can be used to bias reactivation in favor of the consolidation of vocabulary learning (Schreiner & Rasch, 2015a, 2015b), the acquisition of motor skills (Antony et al., 2012), the solidification of emotional memories (Cairney et al., 2014), and the strengthening of object-location associations (Oudiette et al., 2013). Thus, sleep TMR can be considered a relatively robust phenomenon.

However, while TMR has been widely shown to enhance memory when it is applied during sleep, studies exploring the beneficial utility of TMR interventions administered during wakefulness have reached little consensus (see Table 1). On the one hand, evidence from a small number of studies supports the claim that awake-TMR leads to enhancement of subsequent memory. For example, Oudiette et al. (2013) found that auditory cuing of object-locations information during both sleep and wakefulness saved memory for low-value items in a spatial memory task (Oudiette et al., 2013). However, a slightly larger number of studies report null effects of TMR (i.e., no difference in memory performance for cued vs. uncued items). For instance, in a study examining the effect of TMR on memory for Dutch vocabulary words in German-speakers, researchers found that auditory cueing during both passive and active quiescence had no effect on participants’ memory (Schreiner et al., 2015). Finally, still other studies have shown that associative cueing during wakefulness can have a detrimental impact on memory. One example of such a study comes from James et al. (2015), who showed that when emotional memories are recapitulated in an experimental trauma manipulation, followed by an active distractor task, traumatic intrusions were abolished.

**Table 1:** Studies of awake targeted memory reactivation (TMR)

Study	N	Study material	Reactivation manipulation	No. of cuing events	Delay	Effect on memory
Antony et al., 2012	N = 16	Procedural melody learning	Auditory associates (Cover task: working memory)	20 repetitions	~90 min	(0) effect
Cousins et al., 2014	N = 16	Procedural motor learning	Auditory associates (Cover tasks: number comparison)	12 repetitions	8 h	(0) effect
Diekelmann et al., 2011	N = 12	Object- location pairs	Olfactory associates (Cover task: easy motor task)	20-min odor exposure	30 min	(-) effect
Farthouat et al., 2017	N = 41	Word-pairs; mirror tracing	Auditory associates (Cover task: none)	Four repetitions	Immediate and 90 min	(+) effect when word pairs were identical; (-) effect for interference words
Lehmann et al., 2016	N = 21	Word-picture pairs	Auditory associates (Cover Task: Working memory)	Variable (80 min cuing interval)	Immediate and 3 h	(-) effect
Oudiette et al., 2013	N = 30	Objects-location grid pairs	Auditory associates (Cover task: watched a film or working memory)	10 repetitions	30 min and 95 min	(0) effect in one condition; (+) effect In the other
Rasch et al., 2007	N = 18	Object-location pairs; finger tapping	Olfactory Associates (Cover task: Vigilance task)	60 min interval (30-s odor on/off)	8 h	(0) effect
Rudoy et al., 2009	N = 12	Object-location grid pairs	Auditory associates (Cover task: Continuous reaction time task)	Three 7.5-min runs of the task, with embedded sound cues beginning 1.5 min after the start if the middle run	Immediate and 30 min	(0) effect
Salfi et al., 2019	N = 39	Procedural motor learning	Auditory associates (Cover Task: Short Video)	10 min (3 blocks)	10 min	(+) effect
Schreiner & Rasch, 2015a, 2015b	N = 34	Foreign vocabulary words	Auditory associates (Cover task: variety of different tasks)	90 min (10–11 repetitions)	3 h	(0) effect
Schreiner & Rasch, 2015a, 2015b	N = 32	Foreign vocabulary words	Auditory associates (Cover task: variety of different tasks)	90 min (10–11 repetitions)	3 h	(0) effect
Schönauer et al., 2014	N = 29	Finger tapping Sequence	Auditory associates (Cover task: variety of different tasks)	Continuously over 2 h, 1-s rhythmic pace	Immediate and 4 h	(0) effect
Tambini et al., 2017	N = 23	Object-grid location pairs	Visual associates (Cover task: Lexical decision)	Three repetitions, 50-ms visual exposure	Immediate and 24 h	(+) effect
**Present study**	N = 40; N = 40; N = 41	Foreign vocabulary words	Auditory associates (Cover task: Tetris)	36 min (six repetitions)	Immediate and 24 h	(-) effect; (0) effect; (0) effect

While there are several plausible explanations for these discrepant findings, it is notable that most studies exploring awake-TMR have used small sample sizes and include only a singular experiment (i.e., they do not internally assess the robustness or replicability of their findings). Inspection of Table 1 also reveals that across studies, there has been wide variation in the choice of tasks and stimuli, which makes it difficult to know whether something about the stimuli, the reactivation manipulation, the cover task, or some other confounding variable leads to a particular pattern of results.

In the current investigation we addressed these discrepancies and limitations by testing participants across three experiments using similar tasks, TMR manipulations, and stimuli, implementing design improvements and parametric manipulations. In Experiment 1, TMR was administered during an awake-rest phase that followed an initial English-Japanese vocabulary encoding session. The post-encoding period was filled with an easy task, playing the video game Tetris, that kept participants from active rehearsal while also being fun and orthogonal to the encoding paradigm. To span the timeframe of consolidation effects, we tested memory immediately following the cuing phase and after a 24-h delay. In Experiments 2 and 3 we sought to replicate and extend the findings of Experiment 1 by administering similar tasks, but with a few minor changes to the encoding phase (e.g., number of times each associate pair was repeated), the protocol sequence, and the inclusion of comparison conditions. While we initially anticipated that Experiment 1 would replicate recent reports of positive awake TMR effects, the failure to obtain that effect led us to adopt alternative hypotheses in subsequent experiments.

## Experiment 1

In Experiment 1, participants first learned Japanese word meanings according to their English equivalent, and each associated Japanese-English pair was presented along with a sound and image that reflected the definition of each word. For example, for the Japanese word “neko”, meaning “cat”, the Japanese term was shown with the English definition, along with a color image of a cat while a meowing sound was played in the background. Following the initial learning, participants were then asked to play a 36-min round of the game Tetris, which was chosen to serve as a relatively cognitively demanding task that would be entirely orthogonal to (unrelated to) the encoding paradigm. During the Tetris game, half of the learned Japanese-English word pairs were targeted for reactivation by replay of the associated sound (i.e., the semantically related sounds served as the TMR cue). The other half of the vocabulary pairs were left uncued. Participants then completed a recognition test on all learned word pairs – determine whether a presented Japanese-English word pair is correctly matched – both immediately after the TMR manipulation and after a 24-h delay. We hypothesized that Japanese words that were cued during the awake rest phase would be remembered better, especially at the 24-h delay test due to the presumed TMR benefits for consolidation, which are thought to be compounded by sleep.

### Methods

#### Participants

An a priori power analysis was conducted using G*Power version 3.1.9.6 (Faul et al., 2007) to determine the sample size to detect meaningful TMR effects. Based on the effect sizes reported in a meta-analysis for sleep TMR by Hu et al. (2020), we presumed a small to moderate effect size of f = 0.30, α = 0.05, and power = 0.95. Our power analysis was tested on the basis of repeated measures ANOVAs with within-between interactions. On the basis of this power analysis, the minimum sample size needed was N = 40 participants.

Participants were recruited from Temple University’s undergraduate population and the surrounding Philadelphia community. Informed consent was obtained from all participants. Individuals who had a background in Japanese language learning, or who were fluent in Japanese, were excluded from the study. Participants were compensated at a base rate of $15 per hour or were awarded course credit through Temple’s SONA Systems portal.

In Experiment 1, 48 English-speaking participants between the ages of 18 and 30 years (27 female, mean age: 21.4 years) were tested. All participants received an additional compensatory payout based on their performance at immediate and delayed memory testing. Eight participants were excluded due to non-compliance, English being their second language, experimenter error, or being chance responders. These exclusions resulted in a final sample of 40 participants (20 female, mean age: 21.3 years).

#### Materials and design

##### Value manipulation

We incorporated a value manipulation based on a prior study by Oudiette et al. (2013), which demonstrated that awake TMR benefits saved low-value words from forgetting, whereas high-value words were more likely to be consolidated and remembered even in the absence of TMR. Following the same reward procedure as was used in Oudiette et al. (2013), we assigned a low- (1 or 2 points) or high-value (8 or 9 points) number to each of the 72 Japanese nouns used in the foreign vocabulary-learning task. Participants were told that these values would be multiplied by $0.02 to determine the compensatory bonus that they would receive for correctly remembering a given word meaning upon later memory testing. Reward was manipulated in this way to incentivize memory for certain words more strongly than others, setting the stage for the TMR intervention.

##### Encoding phase (see Fig. 1)

**Fig. 1:**
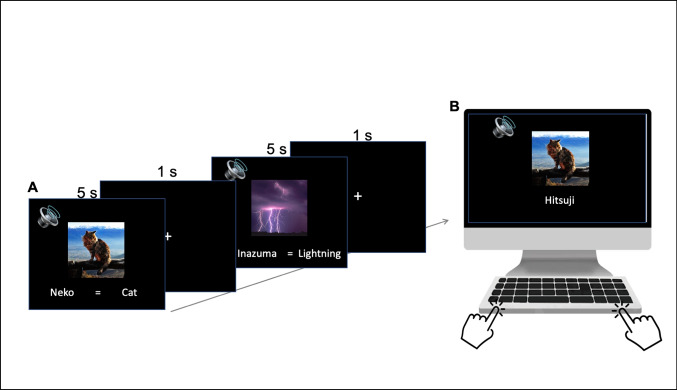
(A) Encoding phase: Japanese vocabulary words and their English translations were presented with corresponding images, for instance a cat, and sounds, for instance “meow.” All 72 words were presented randomly three times over in Studies 1 and 2, and six times over in Study 3. (B) Recognition test: Participants indicated via keypress whether the Japanese word presented to them was congruent or incongruent to the picture shown on the screen

In each trial of the encoding phase, participants were presented with one of 72 neutrally valanced Japanese nouns and its English translation, along with both a descriptive image and a characteristic sound that typified the definition of the noun. Each picture-word-sound triad was onset simultaneously, with the words and picture remaining on the computer screen for 5 s, followed by the presentation of a central fixation cross for a 1-s inter-trial-interval. The 72 triads were sampled randomly and without replacement, broken up into three blocks consisting of 24 trials each, to allow participants to take short breaks across the encoding phase and to thus prevent fatigue. Each block was further divided into six mini-blocks, consisting of four trials in which one word from each of the four possible value denominations was represented. Upon the completion of each mini-block, a display screen would appear with text reading: "Try to maximize your score!" This repetitive message was meant to encourage participants to pay close attention to the values associated with later remembering each word, and as a way to augment the effectiveness of the reward manipulation. After all 72 triads were shown once following this procedure, the procedure was repeated for a second (all 72 triads shown again) and third encoding opportunity. Thus, there were 216 (72 × 3) encoding trials in total.

##### Post-encoding rest and targeted reactivation

Following the completion of the encoding phase, participants began the post-encoding rest phase during which they played Tetris on the computer for 36 min. Tetris was selected as the rest phase task because it is perceptually and conceptually orthogonal to the foreign-language learning task since there are no language demands and it is highly visuospatial. The goal of the rest-phase task was to allow time for consolidation while limiting explicit rehearsal of the vocabulary words, thereby maximizing our ability to make inferences about the unconscious nature of memory reactivations elicited by the TMR procedure. Exactly half of the sounds that had previously been associated with the Japanese vocabulary words were selected pseudo-randomly (based on one of eight random selection schemes that were counterbalanced across subject) and played through a headset, while the other half were held out (never occurred during the post-encoding rest period). The played sounds served as memory reactivation cues for specific words. Each sound cue played repeatedly during a 5-s period. There was a 5-s break between sound presentations. The entire sound playlist was repeated a total of six times, with the 36 selected sounds played in random order for each repetition. Further, it is important to note that the sounds were played at a volume deemed acceptably loud, but not disturbing on a participant-by-participant basis. Subject-specific volume tuning was performed to minimize the level of disturbance that participants may have experienced during the active rest phase (see Whitmore et al., 2022). The appropriate level of cue volume was determined during the practice phase before the official encoding trials ensued, and this volume was kept consistent throughout the rest of the experiment. Additionally, it should be noted that the sounds were played in their raw form and were not embedded in ambient (e.g., “pink”) noise. Finally, the post-encoding reactivation phase lasted 36 min.

##### Memory testing

Immediately after the post-encoding reactivation phase, participants engaged in a recognition memory (“immediate recognition” henceforth) test in which they were presented again with the word-picture-sound triads, only this time without the English translations. Memory was assessed based on participants’ ability to indicate via keypress whether the Japanese word shown on the monitor was semantically congruent with the associated images and sounds that were presented. Participants were instructed to make an “intact” or “mismatch” judgment for the semantically congruent and incongruent triads, respectively. Half of the low- and high-value triads were intact, while half of them were mismatched. To clarify, mismatched trials were created by pairing the images and sounds of unrelated but trained (i.e., previously learned) Japanese words. Recognition task trials were presented in a randomized order. Participants engaged in an identical recognition test 24 h later (“delayed recognition” henceforth).

The trials assigned to cued and uncued and intact versus mismatched were counter-balanced across four different versions of the encoding and testing trials so as to ensure optimal pseudo-randomization of the study procedures. This means that the trials that were mismatched in one version would be intact in another, and the trials that were cued in one version were uncued in another, so that every trial belonged to every condition across iterations of the task.

##### Other assessments

Since participants’ ability to learn foreign vocabulary words may have covaried with general language ability, we used the American National Adult Reading Test (AM-NART) to gauge verbal IQ across all reported studies. Moreover, we asked participants at the end of the study (i.e., at the end of day two after the final memory test) whether they noticed any sounds during the post-encoding rest phase via a SurveyMonkey survey. This was included as a means of ascertaining (1) if the cues entered conscious awareness and (2) whether the cues triggered conscious rehearsal, or whether Tetris was an adequate distractor task insofar as its ability to curb active rehearsal.

#### Statistical analyses

Memory performance was indexed based on mean corrected recognition, in order to control for the trade-off between hit and false alarm rates. Corrected recognition scores were calculated by subtracting the proportion of false alarms from the proportion of hits, calculated separately for each experimental condition. In initial mixed-effects linear regression models, the effects of TMR condition (cued vs. uncued), value (high vs. low), and their interaction, on corrected recognition for the immediate and delayed tests were examined. Since the value manipulation had no impact on performance (see *Results*), subsequent analyses collapsed across different value trials.

After collapsing across low- and high-value trials, we tested a mixed effects linear regression model to assess the interaction between TMR condition (cued vs. uncued) and test day (immediate vs. delay) for corrected recognition. Finally, we re-tested our model to investigate potential differential effects of cuing in low versus high performers, based on a median split of corrected recognition scores. To clarify, the median split was performed by ranking participants based on their corrected recognition scores collapsed across value, and operationalizing low and high performers based on those who fell below or above the median for corrected recognition. This process was performed separately for the immediate and delay time points.

All statistical analyses were performed in RStudio version 4.2.3 (http://www.R-project.org/). All linear mixed effects regression analyses were executed using the *lmer* function from the *lme4* package in RStudio. For all significant interactions, post hoc pairwise comparisons were tested using the *emmeans* package and adjusted using Tukey’s HSD.

### Results

#### Corrected recognition by value

At immediate testing, there was no significant main effect of value (*β* = 0.042, *SE* = 0.033, *p* = .200) or TMR condition (*β* = -0.058, *SE* = 0.033, *p* = .079) on corrected recognition. Additionally, the interaction between value and TMR condition was not significant (*β* = -0.010, *SE* = 0.047, *p* = .828). Similarly, there was no significant main effect of value (*β* = 0.0295, *SE* = 0.036, *p* = .414) or TMR condition (*β* = -0.063, *SE* = 0.036, *p* = .081) on corrected recognition at the delayed test. The interaction between value and TMR condition was also not significant at delayed recognition (*β* = -0.042, *SE* = 0.051, *p* = .414).

#### Corrected recognition collapsed across value

**Fig. 2:**
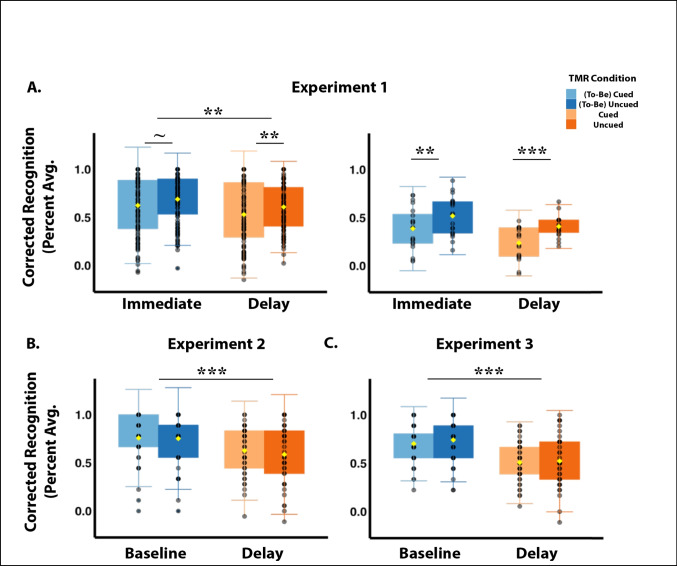
(**A**) Study 1 memory performance for the full sample (left) and for the low performers (right); (**B**) Study 2 memory performance; (**C**) Study 3 memory performance. Note that the legend labels (to-be) cued/uncued points to the fact that in Experiment 1, immediate test, items were already cued, whereas in Experiment 2 and 3, baseline test, participants did not yet know these items were to be cued. Yellow diamonds represent the mean of the distribution. **p < .01, ***p < .001

Summary findings are presented in Fig. 2. Given that we did not find any significant effects of value condition for either test time, we next conducted a model testing TMR condition (cued vs. uncued) by test day (immediate vs. delay), collapsing corrected recognition scores across value conditions. When collapsed across values, a significant main effect of TMR condition emerged wherein cued words were recognized at a *lower* rate than were the uncued words (*β* = -0.066, *SE* = 0.026, *p* = .012). As expected, corrected recognition was also lower at the delayed test compared to immediate testing, regardless of cueing condition (*β* = -0.082, *SE* = 0.026, *p* = .002). The interaction between TMR condition and test day was not significant (*β* = -0.018, *SE* = 0.037, *p* = .626). Subsequent post hoc pairwise comparisons revealed a trending effect of TMR condition at immediate testing (*β* = 0.066, *SE* = 0.026, *p* = .059), and a significant effect of TMR condition at delay testing (*β* = 0.084, *SE* = 0.026, *p* = .009), where at both testing times the uncued words were remembered better than the cued words.

#### Low versus high performers

Considering that cuing was negatively associated with corrected recognition performance, we wondered if this effect might be driven by individuals whose memory performance was low overall. Accordingly, we performed a median split on the data for corrected recognition and ran regression models testing TMR condition (cued vs. uncued) as a function of performance (low vs. high), separately for the immediate and delayed tests. For immediate testing, we found a significant main effect of TMR condition (*β* = -0.132, *SE* = 0.038, *p* < .0001), a significant main effect of performance (*β* = 0.340, *SE* = 0.052, *p* < .0001), and a significant interaction between TMR condition and performance (*β* = 0.132, *SE* = 0.052, *p* = .015). Subsequent post hoc analyses revealed that the low performers remembered uncued words better than cued words (*β* = 0.132, *SE* = 0.038, *p* = .005), while the high performers did not show any memory difference for cued vs. uncued words (*β* = -0.0004, *SE* = 0.037, *p* = 1.00).

Similarly, for delay testing, we found a significant main effect of TMR condition (*β* = -0.172, *SE* = 0.036, *p* < .0001), a significant main effect of performance (*β* = 0.377, *SE* = 0.048, *p* < .0001), and a significant TMR condition by performance interaction (*β* = 0.168, *SE* = 0.049, *p* = .002). Subsequent post hoc analyses revealed that, again, the low performers remembered uncued words better than cued words (*β* = 0.172, *SE* = 0.036, *p* = .0001), while the high performers did not show any memory difference between cued and uncued words (*β* = 0.004, *SE* = 0.034, *p* = .999).

Given the prominent effects in the low performers, we reran the original TMR condition by Test Day interaction model within the subsample of the low performers. This model revealed a significant main effect of TMR condition (*β* = -0.132, *SE* = 0.038, *p* = .001), and a main effect of test day (*β* = -0.096, *SE* = 0.040, *p* = .019). There was also not a significant interaction between TMR condition and test day (*β* = -0.040, *SE* = 0.055, *p* = .470). Post hoc analyses showed a significant effect of cueing at both immediate (*β* = 0.132, *SE* = 0.038, *p* = .006) and delayed testing (*β* = 0.172, *SE* = 0.039, *p* < .001) for the low performers, where uncued words were remembered better at both tests. This suggests that the results low performing participants drove the negative effects of TMR cueing that were observed in this experiment.

Finally, in assessing whether participants noticed the cues during the post-encoding rest phase, we calculated the count and percentage of participants who reported noticing the sounds. Of the 40 participants in this experiment, every subject reported that they heard sounds while playing Tetris; though we did not ask explicitly what they were thinking about during the TMR phase, five participants (12.5%) offered the information that they started to tune out the sounds after a period of habituation.

## Experiment 2

In Experiment 1, we unexpectedly found that awake TMR harmed memory for newly learned foreign language vocabulary words. These effects were driven primarily by the low performers in the cohort. We additionally failed to replicate the value manipulation of Oudiette et al. (2013), who found that awake TMR benefits only occurred for high-value, as compared to low-value, items. These findings prompted us to conduct a replication study. To address some limitations and potential confounds of Experiment 1, we implemented four changes to the Experiment 2 procedure. First, Experiment 1 did not include a true baseline measure of item memory since testing for recognition occurred only after the 36-min rest phase and associated TMR manipulation. Accordingly, in Experiment 2, for half of the vocabulary items, we implemented a “baseline” test of recognition that preceded the TMR manipulation. Second, in Experiment 1, testing of all items at both immediate and delayed recognition may have resulted in testing (retrieval practice) effects that could have obscured delayed (post-consolidation) benefits of the TMR manipulation. Accordingly, a subset of the item triads was held out for testing at only the delayed test. Third, given that we did not find any significant effects of the reward value manipulation in Experiment 1, there was no manipulation of value in Experiment 2. Moreover, we increased the encoding repetitions from three to six to enhance memory strength. Previous memory reactivation research has suggested that underlying representational strength may differentially interact with replay effects in subsequent memory (e.g., Schapiro et al., 2018). Accordingly, we reasoned that the disruptive effects of TMR we observed in Experiment 1 might be explained if participants were only able to form weak representations of item association (Japanese word meaning) with only three presentations of each triad during encoding. Thus, in Experiment 2, we sought to determine whether increasing the strength of memory representations formed during encoding would yield a TMR effect on subsequent recognition in the positive direction. While we acknowledge that the sleep TMR literature suggests that TMR effects are largest for weakly encoded information (Cairney et al., 2016; Creery et al., 2015), these effects are in the opposite direction of those in Experiment 1, wherein we observed a memory decrement due to cuing. Therefore, due to this unexpected finding, we hypothesized perhaps the nature of awake TMR may be different from that implemented during sleep. To see if we could use TMR to bias memory in a positive direction, we wanted to examine if strengthening the underlying memory trace would lead to a more beneficial impact of cuing on memory. We hypothesized that increased exposures to the associate triads during encoding would allow participants to learn the Japanese words more effectively, potentially reducing potential disruptive impacts of the TMR intervention. This hypothesis was informed by the Tambini et al. (2017) study wherein participants were trained to criterion on an object-location task, and for whom memory was improved using a brief reactivation intervention.

Finally, though this is a minor change not affecting the paradigm or intervention directly, we included a question in the end-of-study questionnaire inquiring not only as to whether participants noticed the sounds, but what they were thinking about during the Tetris phase. This was meant to more explicitly ascertain whether participants were consciously rehearsing the words in response to the cues, and if Tetris was demanding enough of a distractor task.

### Method

#### Participants

A total of 50 English-speaking participants between the ages of 18 to 30 years were recruited for the experiment. Ten participants were excluded due to experimenter error or software malfunction. The final sample therefore included a total of 40 participants (26 female; mean age: 21.2 years).

#### Design

##### Encoding phase

Two changes were implemented at the encoding: (1) the triad presentations were increased from three to six times to increase memory strength, and (2) the value manipulation was removed from the study design.

##### Baseline test

The baseline test and stimuli in Experiment 2 were nearly identical to the immediate test implemented in Experiment 1, with three modifications: (1) a baseline memory test was presented immediately at the conclusion of the encoding phase, rather than following the completion of the TMR rest phase. This change provided a more immediate baseline assessment of memory at the conclusion of the encoding period, and before any time-dependent or TMR-related memory changes might occur. (2) We presented only half (36) of the stimuli at the baseline memory test. By holding out the remaining half of the items for later testing, we could dissociate the effects of repeated testing from a TMR effects recognition performance at delay. (3) Participants had 4 s to respond via keypress, rather than no time limit in Experiment 1.

##### Targeted reactivation phase

The Tetris targeted reactivation phase and counterbalancing was identical to Experiment 1. However, the task order differed slightly, where the TMR intervention phase came after the baseline test in Experiment 2.

##### Twenty-four-hour delay test and other assessments

The 24-h delayed test was identical to that in Experiment 1. Participants had 4 s to respond via keypress. The time limit was imposed in Experiment 2 to prevent participants from thinking too long about the correct answer, and to introduce more variability in subject performance, since the TMR effects were only observed in the lowest performers. All other assessments performed were identical to Experiment 1.

#### Statistical analyses

##### Corrected recognition

Corrected recognition was assessed using the same software and analytical methods as employed in Experiment 1 after collapsing across values. Additionally, in Experiment 2, we conducted an analysis testing TMR (cued vs. uncued) by Repeated Testing (tested vs. untested at baseline) interaction effect on the corrected recognition performance at 24-h delay. This additional analysis was conducted to differentiate between the testing effects from TMR effects in subsequent memory, which were confounded in Experiment 1. We assessed if items that were repeated at both baseline and delay showed any unique interactions with TMR effects in Experiments 2 and 3, as only half of the words were tested at baseline and half were not. This allowed us to determine if TMR impacted the items that were repeated twice (tested at baseline and delay) differently from the items that were not tested at baseline to address if repeated testing may have been a confounding variable.

### Results

#### Corrected recognition

A linear mixed-effects regression revealed no significant main of TMR condition in Experiment 2 (*β* = 0.006, *SE* = 0.037, *p* = .881). However, there was a significant main effect of test day, such that average corrected recognition was lower at the delay test compared to baseline (*β* = -0.165, *SE* = 0.037, *p* < .0001). The interaction between TMR condition and test day was not significant (*β* = 0.013, *SE* = 0.053, *p* = .812) (Fig. 2B).

Considering that the negative effects of TMR observed in Experiment 1 were more prominent in low performers, we explored whether low performing participants in Experiment 2 might uniquely exhibit TMR effects. Similar to Experiment 1, we performed a median split and reran our original TMR condition by test day interaction model within the subsample of low performers. There was a main effect of test day (*β* = -0.21, *SE* = 0.07, *p* = .007) in that memory performance was higher at baseline than the delay condition. Unlike Experiment 1, however, this revealed no significant main effect of TMR condition (*β* = 0.007, *SE* = 0.074, *p* = .925), or a significant interaction between TMR condition and test day (*β* = 0.027, *SE* = 0.101, *p* = .791).

Additional analysis investigating whether the re-testing of words across the two test times (tested at baseline and delay) and TMR had differential effects in the delayed corrected recognition performance did not reveal any main effect of repeated testing (*β* = 0.575, *SE* = 0.355, *p* = .108) or TMR (*β* = 0.125, *SE* = 0.355, *p* = .725), or a significant interaction between them (*β* = 0.075, *SE* = 0.502, *p* = .882). We did not investigate the results of the other assessments due to the lack of TMR effects.

#### End of study survey

Survey results indicated that 87.5% (i.e., 35 out of 40) participants noticed the sounds being played during the Tetris phase. Further, when asked what they were thinking about during the TMR phase, participants’ responses were binned into four categories: indication that their thoughts were unrelated to the study (e.g., thinking about work they had to do later); they were thinking about the words that corresponded with the sounds (not necessarily an indication of rehearsal, but indicative of attending to the encoding phase); they were focusing on playing Tetris only; they were thinking about Tetris in combination with the words they had just learned. Results indicated that 15% (i.e., six out of 40) of participants were thinking about something unrelated to the study, 15% were thinking about the words they had just learned and/or the sounds, 60% (i.e., 24 out of 40) were thinking about Tetris only, and 10% (i.e., four out of 40) were thinking about some combination of Tetris and the words or sounds.

## Experiment 3

In Experiment 2 we failed to replicate the disruptive TMR effects we found in Experiment 1. In Experiment 3, we asked whether increasing the underlying memory strength in Experiment 2 may have indeed prevented the disruptive TMR effects we observed in Experiment 1. To test this, in Experiment 3, we presented each triad for three, instead of six, times. The rest of the design was kept identical to that of Experiment 2. This allowed us to compare whether increasing underlying memory strength from Experiment 1 to Experiment 2 could explain the different results obtained in the two experiments, regardless of the other design changes implemented in Experiment 2. Having refined the task design and analytic approach through the preceding experiments, we preregistered Experiment 3 on the Open Science Framework prior to data collection (https://osf.io/y3pd4).

### Method

#### Participants

A total of 45 English-speaking participants, between the ages of 18 and 30 years, were enlisted for the experiment. Four participants were excluded due to experimenter error, task misunderstanding, or failure to return for the Day 2 (delayed testing) session. The final sample used in analyses therefore consisted of 41 participants (24 female; mean age, 21.1 years).

#### Design

The design of Experiment 3 was almost identical to the design of Experiment 2, with the only distinction being the number of encoding repetitions during the encoding phase. Experiment 3 decreased encoding repetition back to three in order to test whether memory strength might explain the lack of any TMR effects in Experiment 2 compared to Experiment 1.

#### Statistical analysis

The software and analyses used were identical to Experiment 2. Similar to Experiment 2, we conducted an analysis testing a TMR (cued vs. uncued) by Repeated Testing (tested vs. untested at baseline) interaction effect on the corrected recognition performance at 24-h delay.

### Results

#### Corrected recognition

The linear mixed-effects regression model revealed no significant main effect of TMR condition (*β* = -0.039, *SE* = 0.040, *p* = .335). However, there was a significant main effect of test day in that average corrected recognition was lower at the delayed test compared to baseline (*β* = -0.241, *SE* = 0.040, *p* < .0001). The interaction between TMR condition and test day was not significant (*β* = 0.022, *SE* = 0.057, *p* = .698) (Fig. 2C).

Similar to Experiments 1 and 2, we performed a median split on recognition performance and reran the original TMR condition by test day interaction model for only the subsample of low performers. Similar to Experiment 2, this analysis indicated a significant main effect of test day (*β* = -0.355, *SE* = 0.057, *p* < .0001), but no significant effect of TMR condition (*β* = -0.031, *SE* = 0.050, *p* = .536) or a significant interaction between TMR condition and test day (*β* = 0.047, *SE* =0.077, *p* = .549).

Similar to Experiment 2, additional analysis investigating whether the re-testing of words across the two test times (tested at baseline and delay) and TMR had differential effects in the delayed corrected recognition performance did not reveal any main effect of repeated testing (*β* = 0.537, *SE* = 0.377, *p* = .158) or TMR (*β* = -0.220, *SE* = 0.377, *p* = .562), or a significant interaction between them (*β* = 0.293, *SE* = 0.534, *p* = .584). We did not investigate the results of the other assessments due to the lack of any significant TMR effects.

#### End of study survey

Survey results indicated that 100% of participants noticed the sounds being played during the Tetris phase. Further, participants’ responses regarding what they were thinking about during the TMR phase were binned in the same fashion as in Experiment 2. Results indicated that 2.44% (i.e., one out of 41) of participants were thinking about something unrelated to the study, 2.44% were thinking about the words they had just learned and/or the sounds, 78.05% (i.e., 32 out of 41) were thinking about Tetris only, and 17.07% (i.e., seven out of 41) were thinking about some combination of Tetris and the words or sounds.

## Comparisons across studies

Given the differing effects of Experiment 1 versus Experiments 2 and 3, we considered the possibility of cohort-based differences across studies. Through analysis of variance (ANOVA), we investigated whether there was significant difference between age, IQ scores, and both baseline and delay corrected recognition performances for the three experimental cohorts. Our analyses revealed no significant differences in age (*F*(2, 117) = 0.835, *p* = .436) or verbal IQ scores (*F*(2, 116) = 1.129, *p* = .327) between cohorts. There were also no significant differences between day-1 corrected recognition (*F*(2, 117) = 2.145, *p* = .122) or day-2 corrected recognition (*F*(2, 117) = 1.606, *p* = .205) performances between cohorts. Therefore, we conclude that the differences in findings cannot be attributed to differences in cohorts.

## General discussion

While targeted memory reactivation (TMR) has been shown to benefit subsequent memory during sleep, TMR effects during awake rest have been less consistent (Hu et al., 2020). Here, we report three behavioral experiments that further challenge hypothesized benefits of TMR during awake rest. In Experiment 1 we found that participants remembered *uncued* Japanese words better than the cued words, at both immediate and 24-h delayed testing, suggesting that TMR had a small detrimental effect on subsequent recognition. Importantly, this effect was more prominent amongst lower performing participants. Given the unexpected direction of these findings, we considered whether particular aspects of the study design may have biased the findings.

In Experiment 2, we implemented some design changes to control for potential confounding variables we identified in the initial design: the immediate recognition test became a true baseline test, and we held out half of the study list to parse out repeated testing effects from TMR effects at the delayed test. Additionally, we sought to enhance the initial encoding of learned Japanese word meanings by increasing the number of repetitions of each triad during the encoding period. It has been previously suggested that replay of weakly encoded information may be detrimental to subsequent memory insofar as it adds additional noise to underlying representations when there is an attempt to recapitulate them during wakefulness (Tanrıverdi et al., 2023). Specifically, we sought to test if encoding strength eliminated the detrimental effect of TMR that we had observed in Experiment 1. Perhaps due to these changes, Experiment 2 found no effect of TMR on subsequent recognition after a 24-h delay.

In Experiment 3, we wanted to further parse out the unique effects of the changes we implemented in Experiment 2. We thought increasing the encoding strength most likely decreased the disruptive TMR effects observed in Experiment 1, but given that it co-occurred with the other changes implemented in Experiment 2, we sought to address it by changing encoding repetitions, thus underlying encoding strength once again. We reasoned that decreasing the encoding strength should produce disruptive TMR effects similar to Experiment 1, if the underlying encoding strength indeed is a predictor of TMR effects. However, Experiment 3 failed to replicate the disruptive TMR effects of Experiment 1, as we again found that no differences between the recognition of cued and uncued words.

When we set out to conduct these studies, we expected that awake TMR would lead to a small but reliable improvement in memory for the cued words, a prediction that followed from the pattern of findings reported in many sleep TMR studies. Because our results instead showed effects that ranged from a small *negative* effect to no effect at all, we revisited the existing corpus of awake-TMR studies, seeking to identify factors that might explain the mixed pattern of findings. Our inclusion criteria were that experiments included some type of wakeful TMR. We did not include any strictly sleep TMR experiments. Table 1 shows that of the 14 existing studies, only four studies found a positive effect of TMR, and in two of those cases, the effects were limited to specific sub-conditions of the experiment. Farthouat et al. (2017) only found a positive TMR effect when word pairs were identical, while Oudiette et al. (2013) only found a positive TMR effect for low-value items. It is difficult to identify any one study feature that distinguishes these four studies from the others reporting null or negative TMR effects – they differ from each other in the types of stimuli tested, the number of repetitions, and the delay before testing, and are undifferentiated from the larger corpus of studies along these same dimensions. Most studies in Table 1 reported no effect of awake TMR on subsequent memory. Thus, the direction of our findings in Studies 2 and 3 are in line with most prior findings. Even though our study used a larger sample size and included two partial internal replication attempts, we were still unsuccessful in finding a positive benefit of TMR. Overall, our findings are consistent with the observation made in a recent meta-analysis that TMR memory benefits are more reliably observed during sleep than awake rest (Hu et al., 2020).

## Limitations and future directions

One limitation that is worth discussion is why we were unable to replicate the value manipulation adapted from Oudiette et al. (2013). That is, why is it that we were unable to use this monetary reward manipulation in order to bias memory towards high-value words, in an effort to save the more weakly encoded low-value words from forgetting using TMR? We conjecture that for some reason, the monetary incentives ($0.08–$0.09 for remembering high-value words, offering a $0.06–$0.08 difference in monetary payout compared to low-value words) was not adequately motivating in our cohort. Future investigations should seek to combat this shortcoming by offering higher financial payouts for the high value items (e.g., perhaps $1.50–$2.00 for high-value words, and $0.25–$0.50 for low-value words).

Moreover, why didn’t we see a positive effect of TMR? The first factor to consider is whether some feature of the rest period affected our results. It could be argued that playing Tetris during rest is suboptimal for TMR because it strongly interferes with memory consolidation. Indeed, there are findings in the post-traumatic stress disorder literature showing that playing Tetris following an experimentally induced traumatic memory, attenuates later flashbacks (James et al., 2015). This finding suggests that an active visuospatial task like Tetris can impede one’s ability to solidify memories, which would be consistent with our findings in Experiment 1, but not with the results of Experiments 2 and 3. This argument is further weakened by the fact that several other awake TMR studies (see Table 1) found negative or null effects, even when they used simple motor distractor tasks, working memory tasks, or distractor tasks that, at face value, have little in common with one another or with Tetris.

It is important to note that our finding that the active nature of the post-encoding rest phase hindered memory performance is in line with research stemming from the non-TMR literature on memory consolidation more generally. For example, one study revealed that 30 min of post-learning “offline” (e.g., passive inward reflection) rest and sleep benefit subsequent memory performance in a manner indistinguishable from each other. This effect was observed in contrast to a post-learning memory phase filled with a distractor task eliciting an “online” state (e.g., active outward interaction with the environment), whose engagement predicted worse memory outcomes on a verbal learning task (Wamsley & Summer, 2020). These findings have been replicated and extended to show the memory benefits of offline wakefulness and sleep support memory for both declarative and procedural memory capacities, further suggesting that the neurobiology of sleep itself is not necessary to provide optimal conditions for consolidative processes (Wang et al., 2021). In the current study, we sought to show that memory reactivation is an “offline” process insofar as its effects on memory are not contingent on active rehearsal or remembering of the previously encoded content. However, we may have made the error of hindering the maintenance of a true “offline” cognitive state by introducing an “online” distractor task. Research has shown that humans spontaneously pivot between offline and online attentional states during passive states (Wamsley & Summer, 2020). The best way to maintain this passive offline state without falling into sleep or online attention remains to be elucidated – a challenge that at least in part may have accounted for failures to replicate such offline/online memory effect dichotomies in another study (Tucker et al., 2020).

A second factor to consider is whether the sensory cues being used to trigger the recapitulation of the neural pattern for specific items only induced weak replay. It is possible that we would have seen larger TMR effects, had we induced more robust reactivations, such as if we re-presented the image, sound, and English word. However, such a scheme poses the risk of soliciting conscious rehearsal rather than unconscious reactivation. While our ability to make inferences about the unconscious nature of reactivation phase is limited in Experiment 1 by the lack of self-report on what the participants were thinking about during the reactivation phase, the sweeping consensus between Studies 2 and 3 was Tetris was demanding enough of a task that they were focused primarily on maximizing their scores during the game. Moreover, we could have presented more sound cues during the Tetris period, thus potentially inducing a stronger replay of information. This is an important limitation to consider since compared to the existing literature, the number of TMR repetitions is relatively low in the current study.

A third factor to consider is whether (or not) the auditory cueing induced any reactivation of the memory trace. Given that this was a behavioral study, we cannot be sure as to whether the auditory cuing intervention reliably reactivated the neural trace for the targeted vocabulary items, or what the strength of that reactivation may have looked like. Despite this, however, retrospective end-of-study self-reports support that some level of reactivation may be inferred given that the overwhelming majority of participants noticed the sounds being played during the rest phase (i.e., between 87.50% and 100% of participants across all three studies). These considerations are, of course, accounting for the uncertainty surrounding the directionality of the hypothesized findings. This is highlighted by a recent study in which we found that more spontaneous neural reactivation during quiescence was associated with worse subsequent memory (Tanrıverdi et al., 2023).

Additionally, it is critical that we address the discrepancy between the small negative effect of cuing in Experiment 1 and the null effect of cuing in Studies 2 and 3. Given that this cannot be attributed to differences between the cohorts, perhaps it may be attributed to the relatively higher number of misses imposed by the time constraints in the follow-up investigations during retrieval. While this was imposed to prevent the potential for ceiling effects, it may have had an influence on the relative corrected recognition scores for cued versus uncued items. Therefore, it stands to reason that perhaps detecting targeted memory reactivation effects are better observed in less constrained retrieval periods.

Finally, it is critical to note that the current report is limited by a number of power-related limitations. First, the power analysis used to determine sample sizes across the three studies was based on the effect sizes in the sleep literature in which the findings are more consistent and robust. Although the scope of Hu et al.’s (2020) meta-analysis was circumscribed to the sleep TMR literature we used it as a basis for our power analysis since it is the largest, most comprehensive overview of any TMR methodology to date. Ideally, we would have liked to base our power analysis on the awake-TMR literature, but a comparable meta-analysis does not exist for the awake-TMR literature yet. While it is clear it would have been better to base the current power analysis on findings from the awake literature, our initial position was hinged on the belief that the mechanism at work during wakefulness should not be fundamentally different from that governing sleep-related consolidation. However, this report and a careful overview of the awake TMR literature reveals that the awake TMR effects are weaker and more fragile than those found in sleep. Similarly, we acknowledge that the partition of the sample into two smaller groups (e.g., low and high performers) in Experiment 1 was an approach that led the sub-samples to fall short of the necessary power deemed necessary to detect an effect based on the sleep literature. Therefore, these results should be interpreted with caution as the between-participants approach suffers from the small sample size in the sub-populations.

In sum, the field has not uncovered the optimal conditions under which positive effects of awake TMR can be reliably obtained. Future research should parametrically manipulate different facets of the TMR intervention to see what types of cues, or combination thereof, are most successful in reactivating the neural encoding pattern that underpins the consolidation process. In addition, future awake TMR investigations should recruit much larger sample sizes to account for the fragility and smallness of the prospective effects.

## Summary

Our findings indicate that awake reactivation effects in humans are fragile and the direction of effects is unpredictable. Whenever a memory enters a labile state, the circumstances surrounding it can alter the memory trace such that it is enhanced or degraded. The challenge now is to find the circumstances that can reliably, and reproducibly, predict the direction of findings.

## Data Availability

Data and analysis scripts for all experiments are available at Experiment 3 ’s preregistration page on the Open Science Framework (https://osf.io/y3pd4).
